# Endoscopic resection of polypoid solitary rectal ulcer: A novel first‐line therapeutic strategy using snare‐assisted mucosal and fibrosis resection

**DOI:** 10.1002/deo2.70108

**Published:** 2025-05-08

**Authors:** Mojgan Forootan, Alessandro Repici, Mohsen Rajabnia, Mohammad Ali Karimi, Ali Jahanian, Pardis Ketabi Moghadam, Mahsa Mohammadi, Erfan Ghadirzadeh, Abdorraoof Soudi, Elham Paraandavaji, Sasan Shafiei, Mohammad Reza Zali, Mohammad Tashakoripour

**Affiliations:** ^1^ Gastroenterology and Liver Disease Research Center, Research Institute for Gastroenterology and Liver Diseases Shahid Beheshti University of Medical Sciences Tehran Iran; ^2^ Humanitas University & Humanitas Research Hospital Milano Italy; ^3^ Non‐communicable Disease Research Center Alborz University of Medical Sciences Karaj Iran; ^4^ Non‐Communicable Disease Research Alborz University of Medical Sciences Karaj Iran; ^5^ Cardiovascular Research Center Mazandaran University of Medical Sciences Sari Iran; ^6^ Skull Base Research Center Shahid Beheshti University of Medical Sciences Tehran Iran; ^7^ Department of Gastroenterology Amiralam Hospital, Tehran University of Medical Sciences Tehran Iran

**Keywords:** endoscopic resection, polypoid lesion, SAMFR, solitary rectal ulcer syndrome, SRUS

## Abstract

**Objectives:**

To propose a novel first‐line endoscopic therapy for treating polypoid lesions in solitary rectal ulcer syndrome (P‐SRUS), the rarest and most challenging subtype of SRUS, which encompasses various endoscopic findings including mucosal erythema, superficial or deep ulcers, and polypoid lesions.

**Methods:**

A prospective, single‐arm study was conducted on 56 patients with histologically confirmed SRUS and broad‐based polypoid lesions in the rectum and anal canal. These patients were referred to the Department of Motility Disorders of the Lower Gastrointestinal Tract. The lesions were removed using snare‐assisted mucosal and fibrosis resection. Patients were monitored for clinical and endoscopic responses at 1, 3, 6, and 12 months post‐treatment.

**Results:**

The study observed improvement in clinical symptoms, a complete endoscopic response, and the absence of late complications following endoscopic resection. Endoscopic evaluations revealed no recurrence of lesions in the follow‐up period.

**Conclusion:**

Endoscopic resection using the snare‐assisted mucosal and fibrosis resection method appears to be an effective and safe treatment option for polypoid SRUS. (Clinical Trial Registration Number: IRCT20211101052935N2).

## INTRODUCTION

**FIGURE 1:**
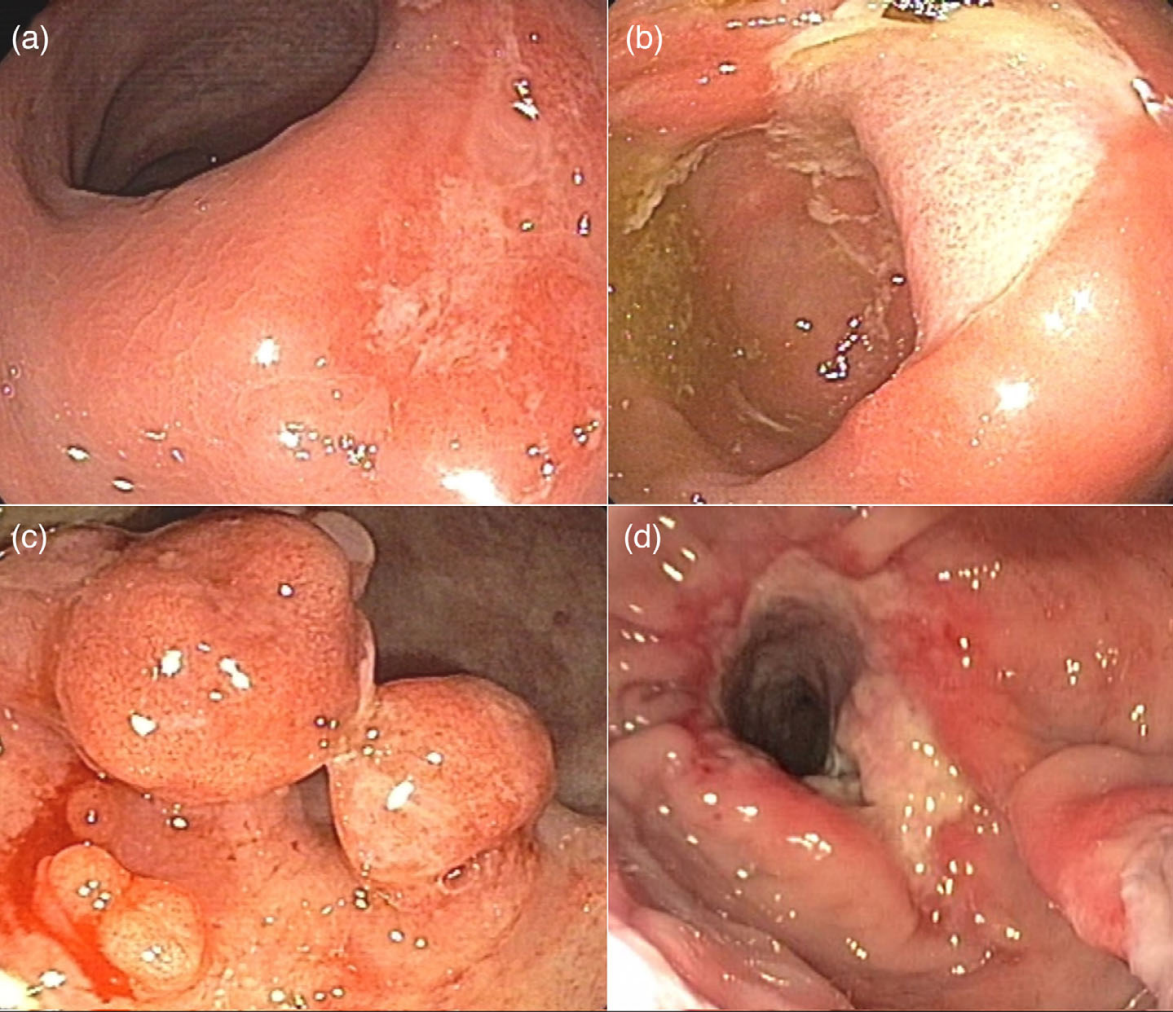
Various types of solitary rectal ulcer syndrome (SRUS): (a) erythema SRUS, (b) superficial or deep ulcers SRUS, (c) polypoid lesions SRUS, and (d) obstructive SRUS.

Solitary rectal ulcer syndrome (SRUS) is a medical misnomer because it includes different types of endoscopic findings, mucosal erythema (E‐SRUS), superficial or deep ulcers (U‐SRUS), polypoid lesions (P‐SRUS), and obstructive SRUS (O‐SRUS), as shown in Figure 1. Consequently, the endoscopic appearance of the disease is often misdiagnosed as rectal cancer or inflammatory bowel disease. Epidemiologic studies have reported an approximate prevalence of SRUS is one in 100,000 people per year. The P‐SRUS prevalence is approximately 17%–25% of these cases.^1^, ^2^, ^3^ From an epidemiological perspective, it does not appear to be gender or age‐specific,^4^, ^5^, ^6^ although it is slightly more common in women, particularly in the third decade of life.^7^, ^8^

Although most patients with SRUS are asymptomatic, several non‐specific clinical manifestations have been attributed to the syndrome, including rectal bleeding, incomplete evacuation, mucous discharge, and a feeling that defecation is blocked.^9^ However, the clinical findings are not pathognomonic for a disease. The treatment of choice is still a challenge as the exact etiology of the disease is not yet known. Dyssynergic defecation, which results from the abnormal contraction of the puborectalis muscle during defecation and eventually leads to local ischemia and ulceration, is the most widely accepted etiology.^10^, ^11^ Patient self‐defecation is also introduced as one of the etiologic factors in the pathogenesis of SRUS. However, the reported inaccessible lesions in the upper rectum and sigmoid colon cannot be justified by digitation, although this association has been rarely reported. Rectal hypersensitivity, resulting in a constant urge to defecate frequent redundant contractions, and mucosal prolapse, leading to mucosal edema, congestion, and blood flow obstruction are also cited as integral components of the pathogenesis of SRUS. Nevertheless, rectal prolapse and SRUS are not always concomitant. The variety of endoscopic features and non‐specific clinical symptoms make the diagnosis difficult. The gold standard for diagnosis is histologic confirmation of hypertrophied muscularis mucosa extending into the evenly distributed crypts in the lamina propria. To determine the pathophysiology of the disease, anorectal manometry, defecography, and electrophysiologic studies are recommended.^12^, ^13^, ^14^

The treatment of SRUS consists of two main components: Elimination of the underlying etiology and improvement of clinical symptoms. If rectal prolapse is not present, a conservative approach is recommended, which includes reassurance, dietary changes, toilet training, biofeedback therapy, and topical anti‐inflammatory agents. Argon plasma coagulation (APC) has been introduced as a successful treatment method for bleeding SRUS. For symptoms that do not respond to conventional therapies, especially rectal prolapse or intussusception, surgical repair is performed, including the Delorme procedure, which is the conventional surgical method for the treatment of P‐SRUS. However, the outcome of surgery is usually suboptimal and complications are not far beyond expectations. Endoscopic therapy is a novel strategy for P‐SRUS. These patients without complete rectal prolapse can be treated with endoscopic methods.

In the current study, snare‐assisted mucosal and fibrosis resection (SAMFR) of polypoid solitary rectal ulcers is presented as a new and minimally invasive treatment strategy for P‐SRUS.

## MATERIALS AND METHODS

### Patient selection

**FIGURE 2:**
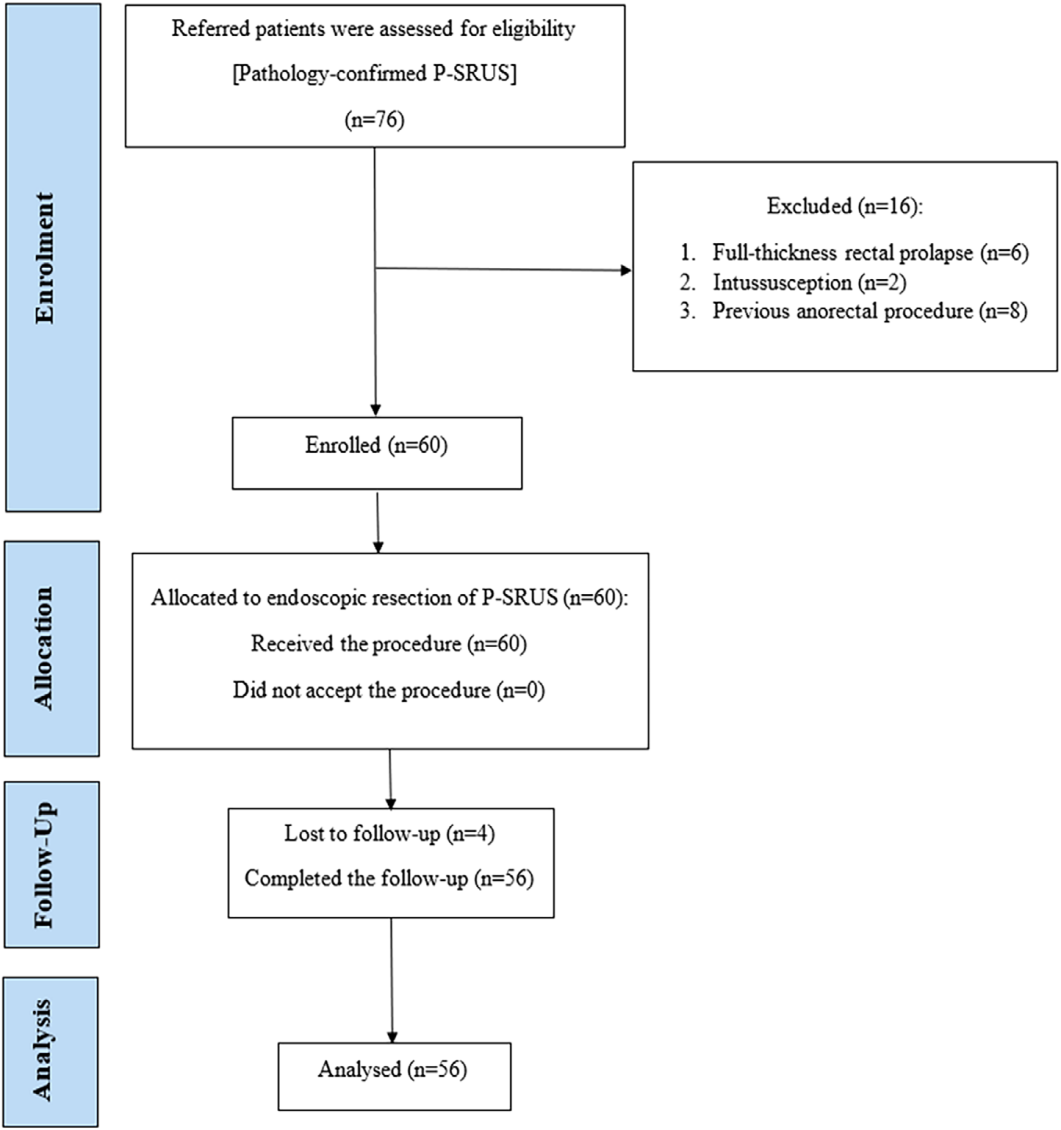
Consolidated Standards of Reporting Trials (CONSORT) flow diagram of the study.

This study prospective, single‐arm study was conducted on patients referred to the Reference Center for Advanced Endoscopic Therapy of Shahid Beheshti University of Medical Sciences, Tehran, Iran, for documented symptomatic SRUS from December 2022 to January 2023 (Figure 2).

All patients with pathologically confirmed P‐SRUS were included in this study. Participants were excluded if they had any of the following: full‐thickness (complete) rectal prolapse, rectal intussusception, severe cardiovascular disease, coagulation disorders, a history of anorectal surgery, or reluctance to participate in the study. Ultimately, 56 patients were included after providing written informed consent.

#### Inclusion and exclusion criteria for endoscopic resection

Inclusion criteria:

- Patients with pathologically confirmed P‐SRUS.
- Lesions amenable to endoscopic resection (ER) are defined as lesions not involving deeper tissue layers or exceeding a manageable size for the technique.

Exclusion criteria:

- Patients with contraindications for ER, including severe cardiovascular disease or coagulation disorders.
- Those with extensive or invasive lesions require surgical resection.
- Reluctance to undergo the procedure or sign consent.

This approach ensured safe and effective treatment while adhering to study protocols.

#### Sample size calculation

The sample size for this prospective study was calculated based on the estimated prevalence of SRUS. Although the exact prevalence of SRUS is not well defined, it has been estimated to occur in approximately 1 in 100,000 people per year.^3^ Considering this rare prevalence, an expected proportion (*p*) of 0.00001 was used for the calculation. The formula for estimating a single proportion with precision was applied:

$$n=\frac{Z^{2}\times p\times \left(1-p\right)}{d^{2}}$$

where 𝑍 is the Z‐value for a 95% confidence level (1.96), and d represents the margin of error, which was set at 10% (0.10). Substituting the values into the formula, the calculated sample size was 1. Given the rarity of the condition and the very low prevalence rate, obtaining a sufficiently large sample size to power the study using this prevalence estimate was not feasible. Instead, a pragmatic sample size of 56 patients with pathologically confirmed polypoid lesions in SRUS was included.

### Intervention

ER was performed to remove polypoid lesions in the anal canal and rectum. A submucosal injection of saline mixed with epinephrine (1:10,000) was administered to lift the lesion and minimize the risk of thermal injury to the deeper layers. A monopolar electrosurgical ERBE VIO3 generator was used, with ER performed using an intermittent high‐frequency cutting cycle followed by a coagulation cycle in Q mode (ENDO CUT Q), with the following settings: effect 4, cut duration 1, and cut interval 6. For hemostasis, soft coagulation was applied with effect 4 and a maximum wattage of 35. The ER was conducted using a 25‐mm oval‐shaped snare for smaller lesions and a 33‐mm crescent‐shaped snare for larger or irregularly shaped lesions. These snares were chosen for their flexibility, precision, and ability to conform to the lesion's contours, facilitating complete resection while minimizing mucosal injury. Additionally, their insulated distal tips helped prevent unintended thermal damage. To prevent stricture, circumferential and recto‐anal lesions were removed in two to three consecutive sessions with a minimum interval of 2 weeks. The resected polyps were retrieved using a polyp retriever net, ensuring care to avoid mucosal injury.

**FIGURE 3:**
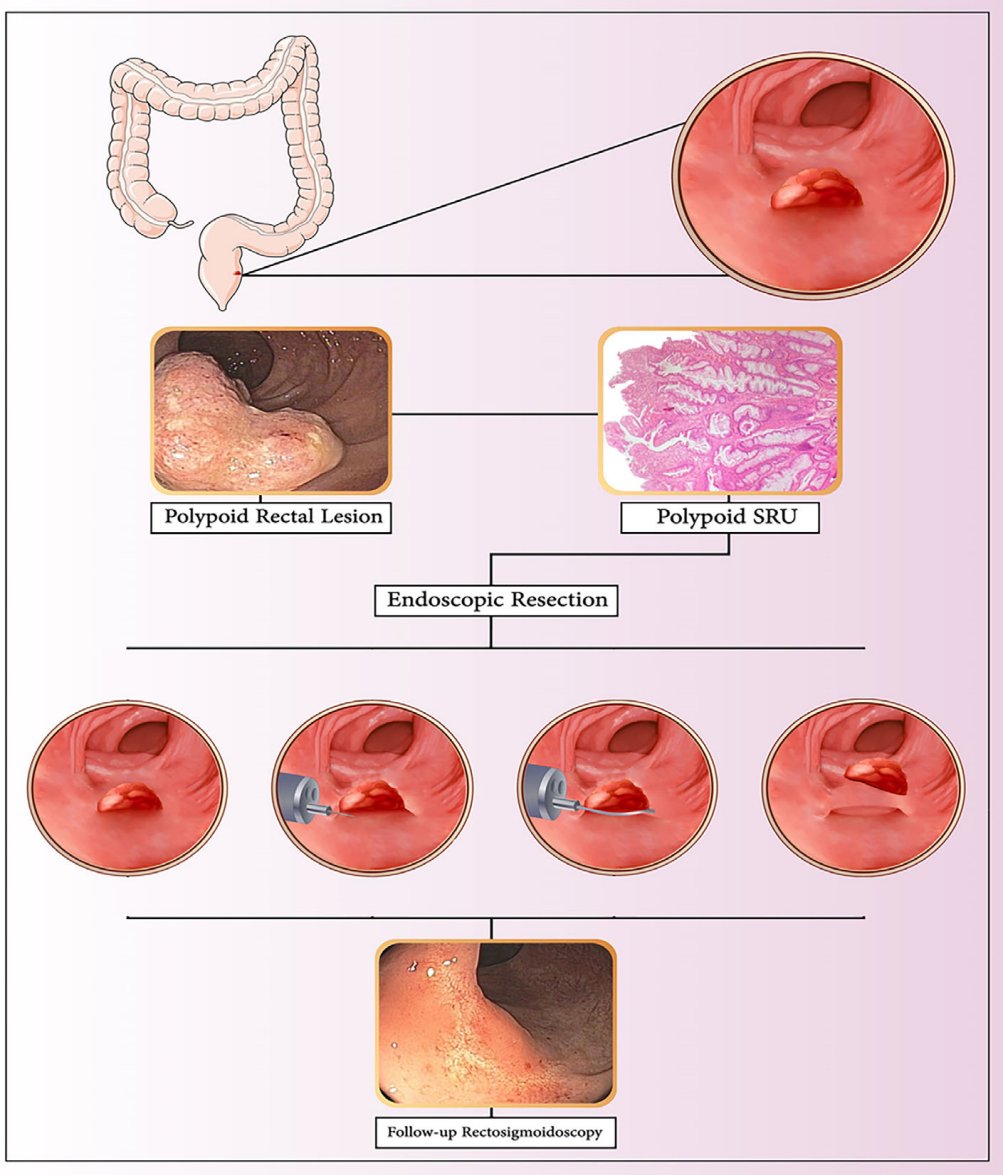
Graphical summary of the ER of a polypoid solitary rectal ulcer.

**FIGURE 4:**
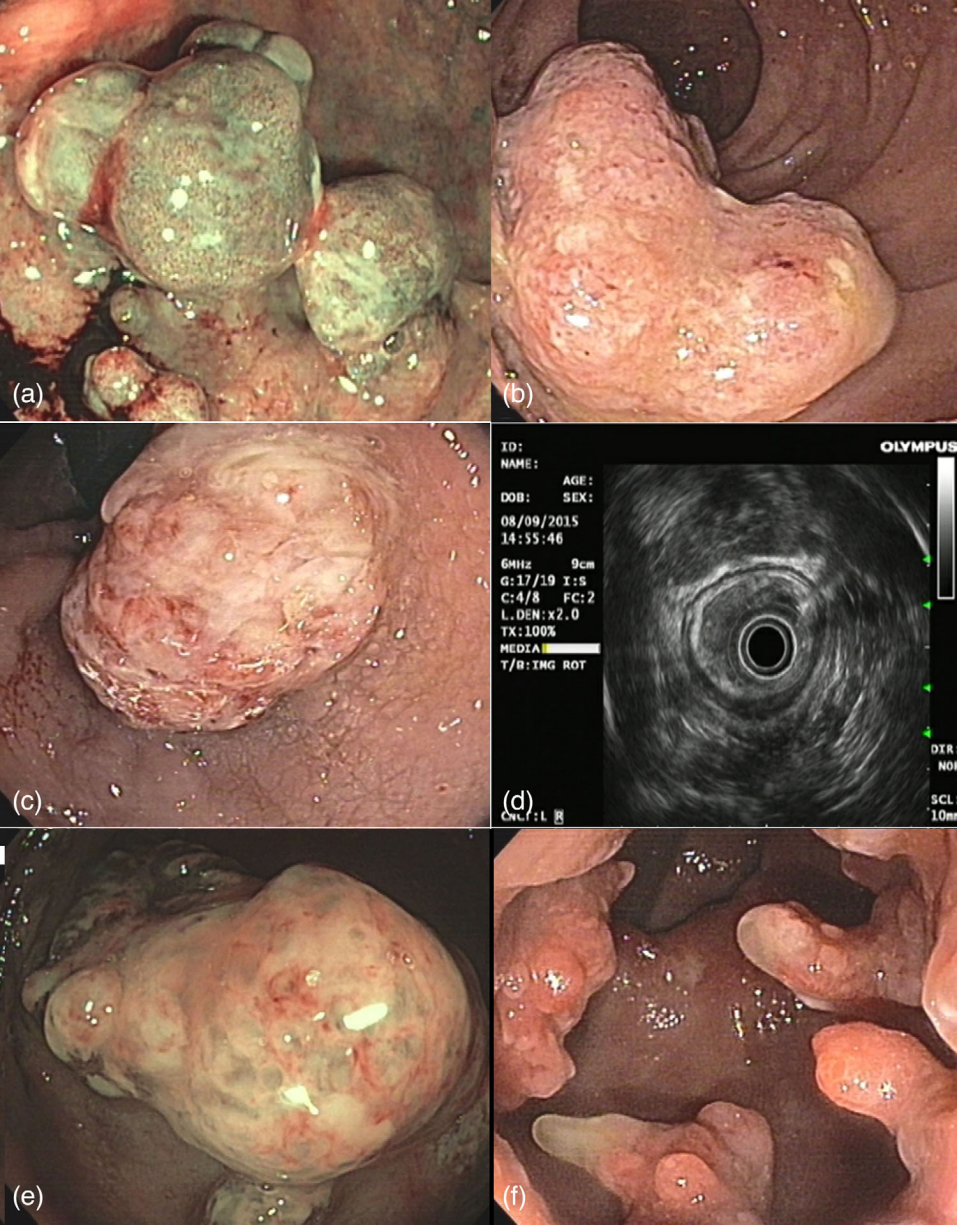
Various presentations of polypoid solitary rectal ulcer syndrome (P‐SRSUS) in the anal canal and rectum: (a, e) Narrow‐band imaging (NBI) views showing the characteristic vascular patterns of polypoid lesions in the rectal and anal canal. These images highlight the altered microvasculature, with capillary loops and irregular vessel distribution indicative of mucosal changes associated with P‐SRSUS. (b, c, f) White‐light endoscopy revealing the surface morphology and color of the rectal and anal canal lesions. Panel (b) demonstrates a large, irregular polyp with a hyperemic surface, while panel (c) shows a nodular lesion with visible mucosal irregularities. Panel (f) presents multiple smaller polypoid formations along the rectal mucosa. (d) Endoscopic ultrasound view displaying the lesion's hypoechoic characteristics and submucosal involvement. This imaging aids in evaluating the depth of invasion and the lesion's relationship with surrounding structures.

The specimens were fixed and well‐oriented before being sent to the laboratory for histological evaluation. After the procedure, patients were monitored in the recovery room for at least 6 h for vital signs, and clinical symptoms indicative of immediate complications such as abdominal pain, local tenderness, and bleeding. Subsequently, clinical symptoms were followed until 48 h after discharge. They were trained in detail about the alarm signs. They were advised to eat soft food for at least 3 days after the procedure. They were also told to avoid manipulation and self‐digestion to avoid direct trauma to the mucous membranes. All patients underwent behavioral therapy and biofeedback regardless of the clinical manifestations. Figure 3 represents a graphic summary of the ER of a polypoid solitary rectal ulcer. Figure 4 represents various presentations of polypoid solitary rectal ulcer syndrome in the anal canal and rectum. 4

### Patient follow‐up

The treatment strategy aimed to control clinical symptoms, including rectal bleeding, mucosal discharge, difficult or painful defecation, digitation, and the feeling of incomplete evacuation. Patients were monitored at regular intervals for symptom improvement and the occurrence of adverse events. All patients were evaluated at 1, 3, 6, and 12 months following the index procedure through a detailed medical history and flexible recto‐sigmoidoscopy. Regular annual follow‐up examinations were also recommended.

Clinical outcomes were categorized as primary and secondary outcomes. Primary outcomes involved improvements in symptoms, while secondary outcomes were defined as follows:

- Complete remission (CR): Defined as the complete disappearance of all symptoms, with no residual signs or evidence of disease during follow‐up.
- Partial remission (PR): Defined as an improvement in the severity and frequency of symptoms compared to baseline, with some residual symptoms still present.
- Non‐responder (NR): Defined as no significant change in the severity or frequency of symptoms compared to baseline, with patients continuing to experience persistent symptoms such as regular rectal bleeding or severe difficulty during defecation.

### Statistical analysis

Categorical variables are expressed as numbers and percentages. For the analysis of categorical variables, the *χ*2 or chi‐square test was used where appropriate. The chi‐squared test was performed to assess the distribution of patients across the three groups over time, from baseline to 1‐, 3‐, and 6‐month follow‐ups. All analyses were performed using the IBM SPSS 27.0. A two‐tailed *p* < 0.05 was considered statistically significant. The decrease in the number of symptoms and findings at colonoscopy after ER compared to before ER was calculated as percentage decrease  =  (*N* after therapy ‐ *N* before therapy)/*N* before therapy.

### Ethical considerations

Patients were enrolled in the present study after providing written informed consent. They could withdraw from the study at any time; nevertheless, they were advised to complete the treatment.

## RESULTS

Of the patients eligible for the current study, a total of 56 patients completed the minimum 1‐year follow‐up of the study and were eventually enrolled in the ongoing study. The baseline clinical characteristics of patients are summarized in Table 1. Most of them were male and in their second to third decade of life. The main focus of the present study was whether or not endoscopic polypectomy could alleviate the signs and symptoms of SRUS. Various presentations of polypoloid solitary rectal ulcer syndrome in the anal canal and rectum in patients are shown in Figure 3.

**TABLE 1:** Baseline clinical characteristics of patients.

Characteristic	Value
**Gender**	
Male	40 (71.4%)
Female	16 (28.6%)
**Age (years)**	
Mean ± SD	28.8 ± 5.4
Range	18–49
**Age distribution (years)**	
18–29	39 (69.6%)
30–39	13 (23.2%)
40–49	4 (7.2%)
**Height (cm)**	163.5 ± 7.8
**Weight (kg)**	71.3 ± 10.2
**Body Mass Index (BMI, kg/m^2^)**	23.7 ± 2.5
**Comorbidities**	
Irritable bowel syndrome	10 (17.9%)
Hemorrhoids	8 (14.3%)
History of anxiety or depression	6 (10.7%)
Hypertension	4 (7.1%)
Diabetes mellitus	3 (5.4%)
**Blood cell counts (mean ± SD)**	
Hemoglobin (g/dL)	13.2 ± 1.4
White blood cells (×10^3^/µL)	7.8 ± 2.3
Platelets (×10^3^/µL)	265 ± 40
Neutrophils (%)	60.5 ± 8.6
Lymphocytes (%)	30.2 ± 6.7

**FIGURE 5:**
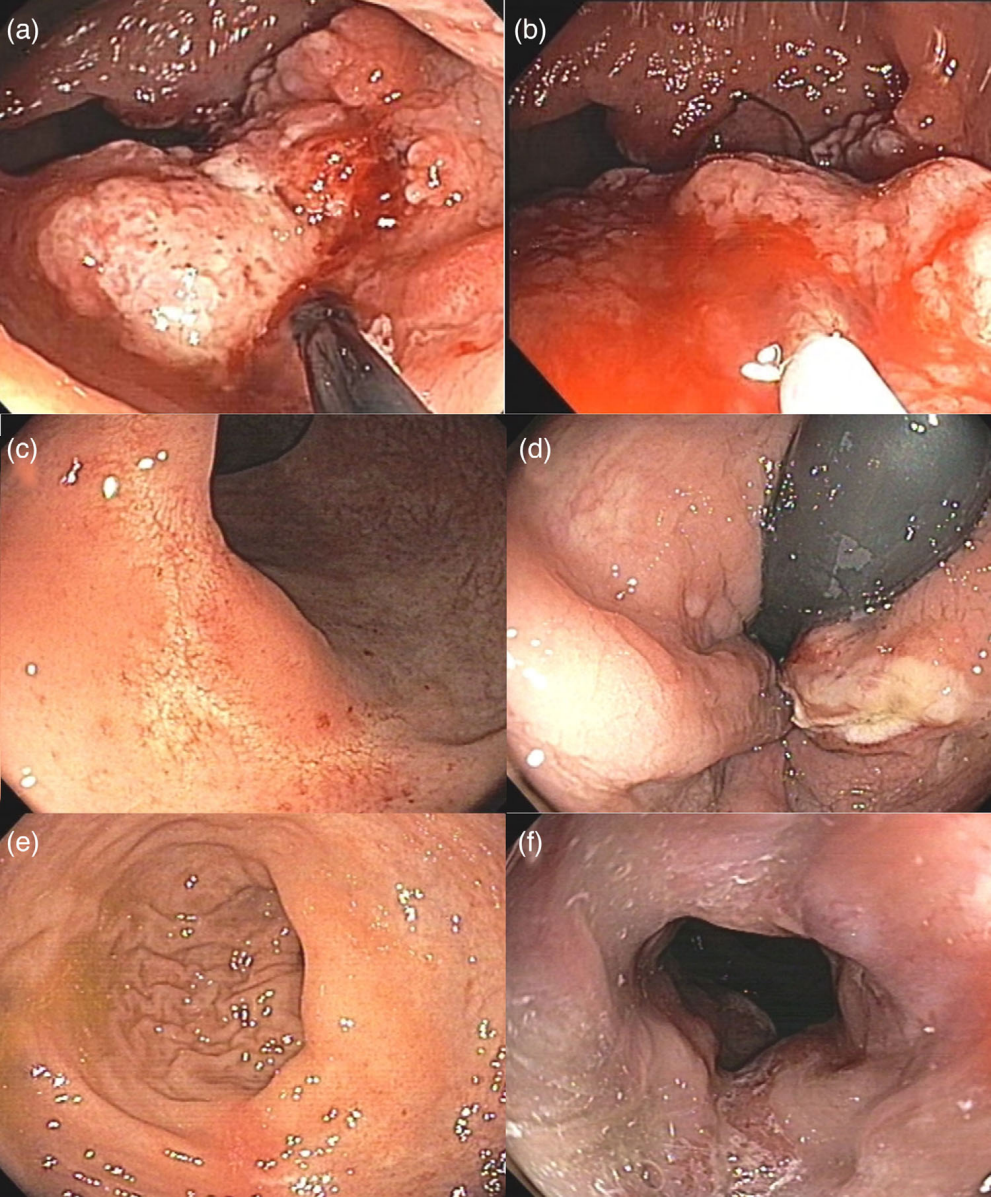
Steps of snare‐assisted mucosal and fibrosis resection (SAMFR) technique and follow‐up: (a, b) SAMFR, (c, d) after resection, and (e, f) follow‐up after 2 months.

The clinical signs and symptoms of the participants before and after the procedure were compared and listed in Table 2. Figure 5 shows the steps of the SAMFR technique and follow‐up. As shown in Table 2, within 1‐month post‐procedure, complete response (CR = 100%) was observed in all patients with rectal bleeding (56/56), self‐digitation (53/53), and mucopurulent discharge (51/51). CR rates for obstructive sensation, difficult defecation, and incomplete voiding were 77.5% (31/40), 40.82% (20/49), and 72.92% (35/48), respectively. The procedure significantly improved clinical symptoms, including rectal bleeding, anorectal obstruction, mucous discharge, self‐digitation, difficult defecation, and incomplete evacuation, at the 6‐month follow‐up (*p* < 0.05).

**TABLE 2:** Participants’ symptoms attributable to solitary rectal ulcer syndrome before and after endoscopic resection.

Clinical manifestation	Presentation		1st F/U (1 month)			CR (%)	2nd F/U (3 months)			3rd F/U (6 months)			*p*‐value	4th F/U (≥ 1 year) (*n* = 49)
	Yes	No	CR	PR	NR		CR	PR	NR	CR	PR	NR		
Rectal bleeding (*n*)	56	0	56	0	0	100	56	0	0	56	0	0	<0.05	No recurrence
Feeling of obstruction (*n*)	40	16	31	9	0	77.5	38	2	0	40	0	0	<0.05	No recurrence
Mucous discharge (*n*)	51	5	51	0	0	100	51	0	0	51	0	0	<0.05	No recurrence
Self‐digitation (*n*)	53	3	53	0	0	100	53	0	0	53	0	0	<0.05	No recurrence
Difficult defecation (*n*)	49	7	20	23	6	40.82	34	15	0	49	0	0	<0.05	No recurrence
Feeling of incomplete evacuation (*n*)	48	8	35	11	2	72.92	42	6	0	48	0	0	<0.05	No recurrence

Abbreviations: CR, complete response; PR, partial response; NR, no response.

2‐ and 6‐month follow‐ups only include patients without complete remission in the previous time interval.

Incomplete evacuation was initially reported by 48 participants, with complete remission achieved by 35 after 1 month, seven after 3 months, and six after 6 months (*p* < 0.05). Obstructive sensation was reported by 40 participants, with remission in 31 after 1 month, seven after 3 months, and two after 6 months (*p* < 0.05). Difficult defecation affected 49 participants, with remission in 20 after 1 month, 14 after 3 months, and 15 after 6 months (*p* < 0.05). Chi‐square tests confirmed significant correlations between polyp resection and symptom resolution for all conditions. Of the 56 patients included in the study, 49 were endoscopically confirmed to have no recurrence after 1 year of follow‐up. The remaining seven patients could not be reached for their annual follow‐up. The annual review of the clinical and endoscopic symptoms of the 49 patients revealed no evidence of recurrence. The findings of the ongoing study suggest that ER of polypoid lesions in symptomatic P‐SRUS can significantly alleviate symptoms attributed to SRUS within 6 months of the procedure. Additionally, no major or minor complications, such as perforation, early or late bleeding, anal pain, or stricture, were observed in this study.

## DISCUSSION

This study evaluated the effectiveness of endoscopic polypectomy in alleviating symptoms of SRUS in 56 patients with a minimum 1‐year follow‐up. The procedure led to significant improvements in symptoms such as rectal bleeding, self‐digitation, mucopurulent discharge, obstructive sensation, and difficult defecation, with complete remission achieved in several patients within 1 month. At the 6‐month follow‐up, significant symptom reduction was observed, and no major complications were reported. These findings suggest that ER of polypoid lesions is a safe and effective treatment for symptomatic P‐SRUS.

SRUS is a rare, benign condition presenting with a range of symptoms, from mild lesions to severe issues like rectal bleeding, defecation disorders, and rectal prolapse. The diagnosis is confirmed through histologic examination, showing muscle fiber extension and fibrosis in the lamina propria. Paradoxical contraction of the puborectalis muscle, leading to high anal canal pressure during defecation, is observed in over 80% of SRUS cases. While the etiology remains unclear, various factors have been proposed. Treatment options, including dietary changes, laxatives, 5ASA enemas, sucralfate enemas, and biofeedback therapy, often have limited efficacy and are primarily short‐term solutions.^15^, ^16^ However, it should be noted that pharmacotherapy has a high failure rate in these patients.^17^ Moreover, adherence to behavioral therapy, which consists of long‐term courses, plays a key role in the effectiveness of therapy.^18^ Another problem with the medical and behavioral approach is that they are only beneficial for a small number of patients and only for a short‐term course.^19^, ^20^ Nevertheless, they can be integrated into other therapeutic methods to improve the effectiveness of the therapy.^16^ The treatment of SRUS includes a spectrum of options ranging from conservative management to surgical interventions, depending on the severity of the condition. Conservative approaches, including dietary modifications, bulk laxatives, and biofeedback therapy, are often effective for patients with mild to moderate symptoms, particularly in the absence of mucosal prolapse.^3^ However, these methods may fail in advanced cases with significant intussusception or fibrosis, necessitating more invasive measures. Topical therapies such as sucralfate, corticosteroid, or sulfasalazine enemas have shown limited efficacy in uncontrolled case series, but their long‐term outcomes remain uncertain.^21^, ^22^

These challenges are prompting physicians to find alternative methods for the treatment of SRUS. Endoscopic treatment is one such new approach that is being proposed alongside other topical treatments such as sucralfate enema, corticosteroid enema, and botulinum toxin.^21^, ^23^, ^24^ New therapeutic methods are increasingly being introduced for the treatment of SRUS, as symptomatic lesions usually do not respond to conservative therapy. Endoscopic APC is a promising method for the treatment of bleeding SRUS (U‐SRUS). Shah et al. and Zergani et al. have demonstrated that APC is an effective endoscopic method of choice for hemostasis, reducing ulcer size and improving tissue repair in SRUS.^25^, ^26^ Long‐term studies are needed to evaluate the long‐term effects of the therapy. To the best of our knowledge and based on our studies, there seem to be different treatment methods for various types of SRUS. Although P‐SRUS is less common than the other types, its treatment is more difficult. To date, the Delorme procedure has been used for the treatment of P‐SRUS. The current study proposes an endoscopic method as a minimally invasive treatment method for the management of P‐SRUS. This treatment can potentially be used as a first choice for targeted cases. Jha et al. reported a case and presented ER of polypoid lesions as an effective method to treat patients who do not respond to medical and APC treatments.^27^ In a case series conducted by Perito et al., four patients underwent polypectomy, which resulted in faster remission than other methods.^28^ The results of ER were also acceptable in a few reported cases around the world.^29^, ^30^ Surgical techniques are usually reserved for cases where treatment fails and rectal prolapse is present. The reported surgical techniques are rectopexy, lesion excision, and Delorme's procedure.^15^, ^16^, ^23^ Ihnat et al. conducted a study on the combination of laparoscopic resection‐rectopexy with transanal endoscopic microsurgery, which proved to be a safe and effective treatment method in complicated cases.^31^, ^32^, ^33^ Potential complications of endoscopic procedures, such as perforation and bleeding were not reported in the present study, with the exception of one patient who experienced immediate bleeding requiring hemostasis using a co‐grasper. As shown in Table 1, rectal bleeding, mucous discharge, and sensation of anorectal obstruction are significantly alleviated by the SAMFR method during the first follow‐up of the participants. However, the evaluation of difficult defecation, sensation of incomplete evacuation, and sensation of obstruction did not indicate satisfactory results by the SAMFR method 1 month after the index procedure, emphasizing the importance of biofeedback therapy as a complementary etiological treatment for immediate response as well as prevention of recurrence in case of such troublesome symptoms of SRUS.

## CONCLUSION

In the current study, the SAMFR method was presented as a novel and successful approach for the first‐line treatment of P‐SRUS. To prevent the recurrence of symptoms, the commonly accepted underlying etiology, that is, dyssynergia and defecation should be controlled by biofeedback and behavioral therapies. It should be borne in mind that this is not an appropriate method of therapy in cases of complete rectal prolapse or intussusception and should not be recommended. In such cases, however, a combination of SAMFR and surgical repair would be appropriate. The present study was subject to several limitations that should be taken into account. First, the single‐arm study design limits the ability to draw definitive conclusions about the observed results as there is no control group for comparison. Additionally, being a single‐center study may introduce biases related to patient demographics, disease characteristics, and treatment practices at each center, potentially limiting the generalizability of the findings to broader populations. These limitations emphasize the need to be cautious in interpreting the results and highlight the importance of future research efforts with more robust study designs and broader patient cohorts to validate and extend the findings.

## CONFLICT OF INTEREST STATEMENT

None.

## ETHICS STATEMENT

The protocol of this clinical trial was approved by the Institutional Review Board of Shahid Beheshti University of Medical Sciences. All procedures were conducted according to the Declaration of Helsinki.

## PATIENT CONSENT STATEMENT

All patients provided signed informed consent before participating in the study.

## CLINICAL TRIAL REGISTRATION

The trial was registered with the Iranian Registry of Clinical Trials (Approval ID: IRCT20211101052935N2).
